# Author Correction: De novo identification of essential protein domains from CRISPR-Cas9 tiling-sgRNA knockout screens

**DOI:** 10.1038/s41467-020-14940-7

**Published:** 2020-02-25

**Authors:** Wei He, Liang Zhang, Oscar D. Villarreal, Rongjie Fu, Ella Bedford, Jingzhuang Dou, Anish Y. Patel, Mark T. Bedford, Xiaobing Shi, Taiping Chen, Blaine Bartholomew, Han Xu

**Affiliations:** 1 Department of Epigenetics and Molecular Carcinogenesis, The University of Texas MD Anderson Cancer Center, Smithville, TX 78957 USA; 2 The Center for Cancer Epigenetics, The University of Texas MD Anderson Cancer Center, Houston, TX 77030 USA; 3 Center for Epigenetics, Van Andel Research Institute, Grand Rapids, MI 49503 USA; 4 Department of Bioinformatics and Computational Biology, The University of Texas MD Anderson Cancer Center, Houston, TX 77030 USA

**Keywords:** Data processing, High-throughput screening, CRISPR-Cas9 genome editing

Correction to: *Nature Communications* 10.1038/s41467-019-12489-8, published online 4 October 2019.

The original version of this article omitted the following from the Acknowledgements:

H.X. is a CPRIT Scholar in Cancer Research.

This has now been corrected in both the PDF and HTML versions of the article.

