# Supplementary material for: De novo identification of essential protein domains from CRISPR-Cas9 tiling-sgRNA knockout screens
Source: Nat Commun. 2019 Oct 4;10:4541. doi: 10.1038/s41467-019-12489-8 (PMC6778102; doi:10.1038/s41467-019-12489-8)
Supplement: Supplementary file 1 — Supplementary Information [file 41467_2019_12489_MOESM1_ESM.pdf]

Supplementary Information

De novo Identification of Essential Protein Domains from CRISPR/Cas9 Tiling-sgRNA Knockout Screens

He et al.

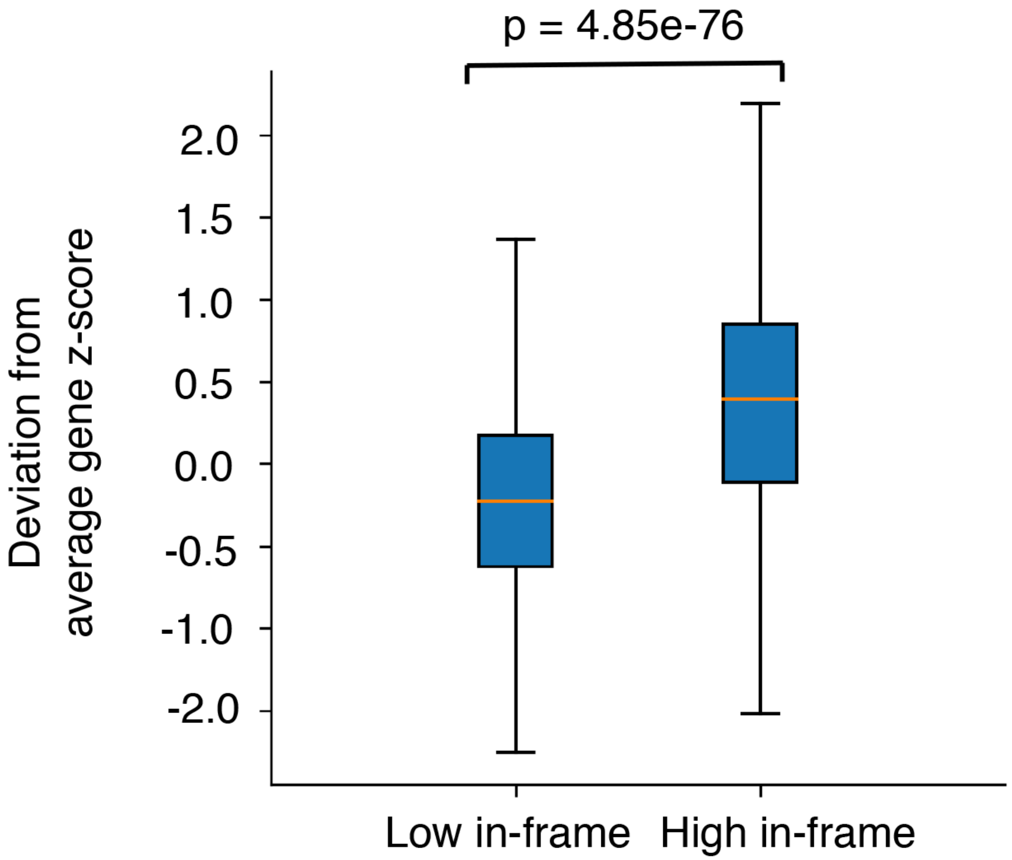

Supplementary Figure 1 Boxplot comparing dropout effect of “high in-frame” and “low in-frame” sgRNAs in Munoz dataset[1]. The p-value was calculated using Mann-Whitney test. The center line, bounds of box and whiskers represent the median, interquartile range and 1.5 times interquartile range, respectively.

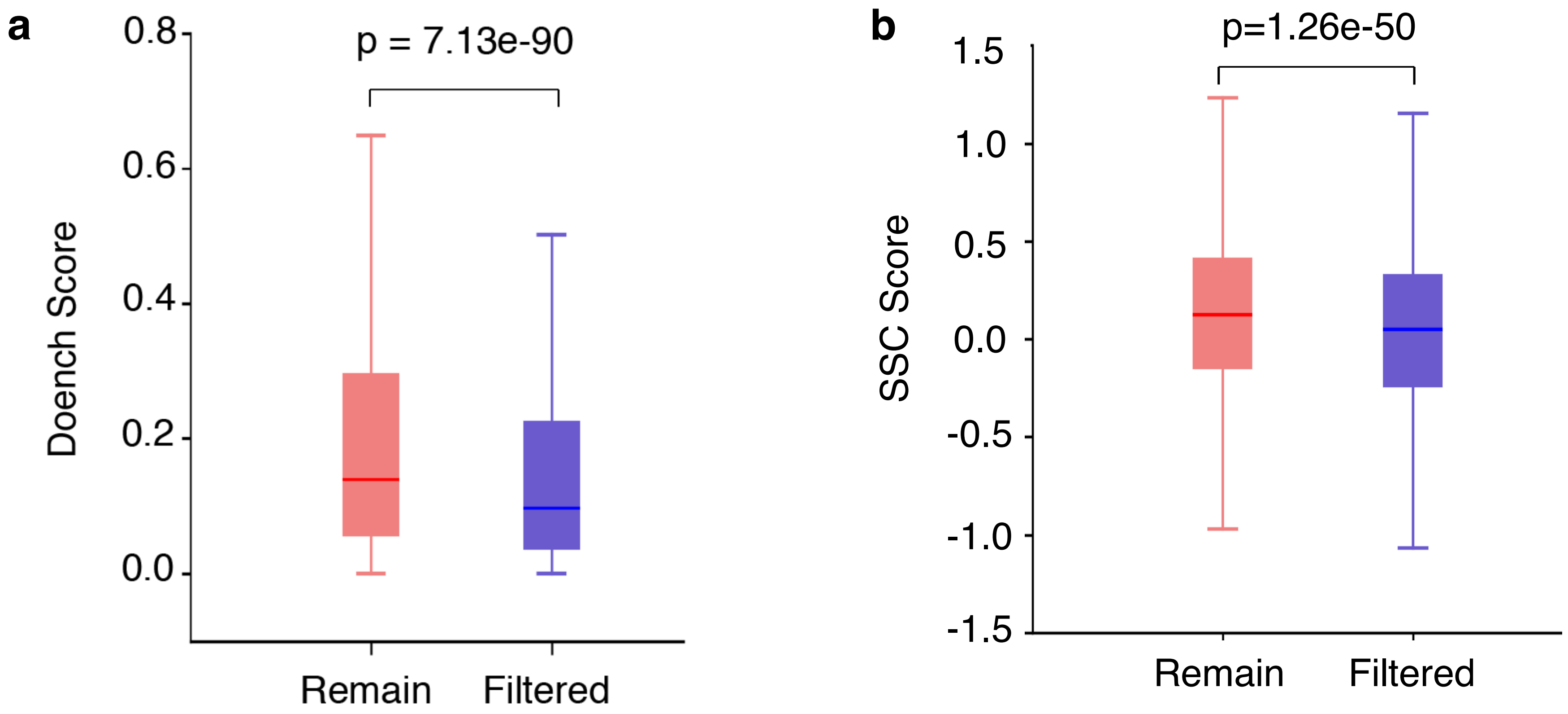

Supplementary Figure 2 Boxplot comparing **a)** Doench scores[2] and **b)** SSC scores[3] of filtered and remaining sgRNAs in the process of weak signal removal. The p-values were calculated using Mann-Whitney test. The center line, bounds of box and whiskers represent the median, interquartile range and 1.5 times interquartile range, respectively.

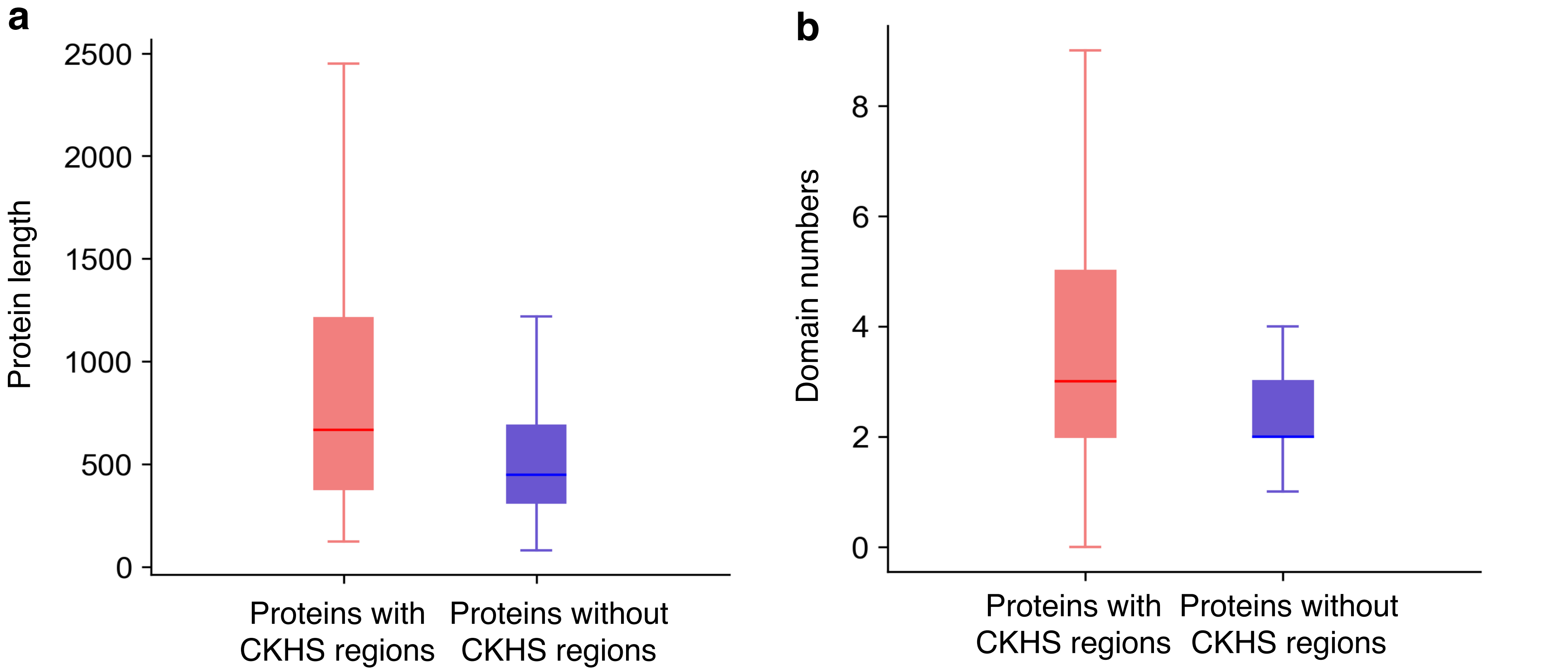

Supplementary Figure 3 a) Boxplot comparing the length of proteins with and without CKHS regions called by ProTiler. **b)** Boxplot comparing the domain numbers of proteins with and without CKHS regions called by ProTiler. The center line, bounds of box and whiskers represent the median, interquartile range and 1.5 times interquartile range, respectively.

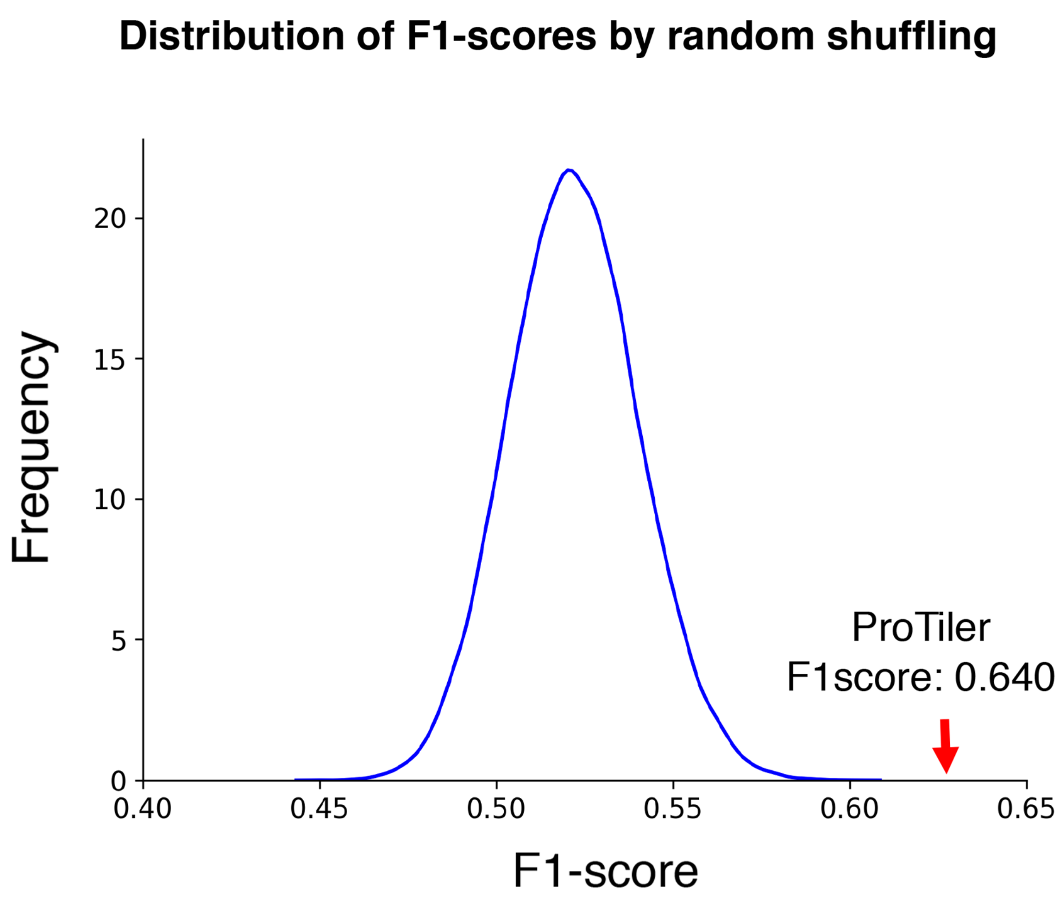

Supplementary Figure 4 Distribution of F1-scores with random shuffled protein regions for detecting Pfam protein domains

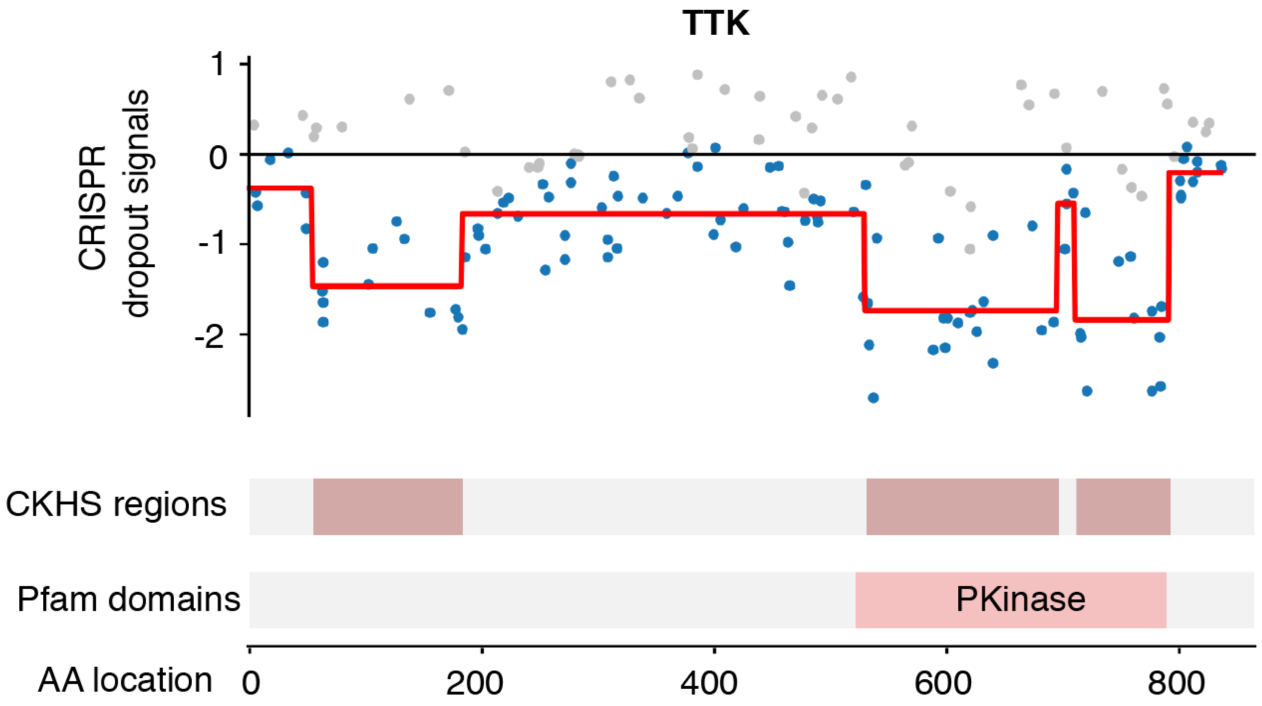

Supplementary Figure 5 CKHS profile and domain annotation of TTK.

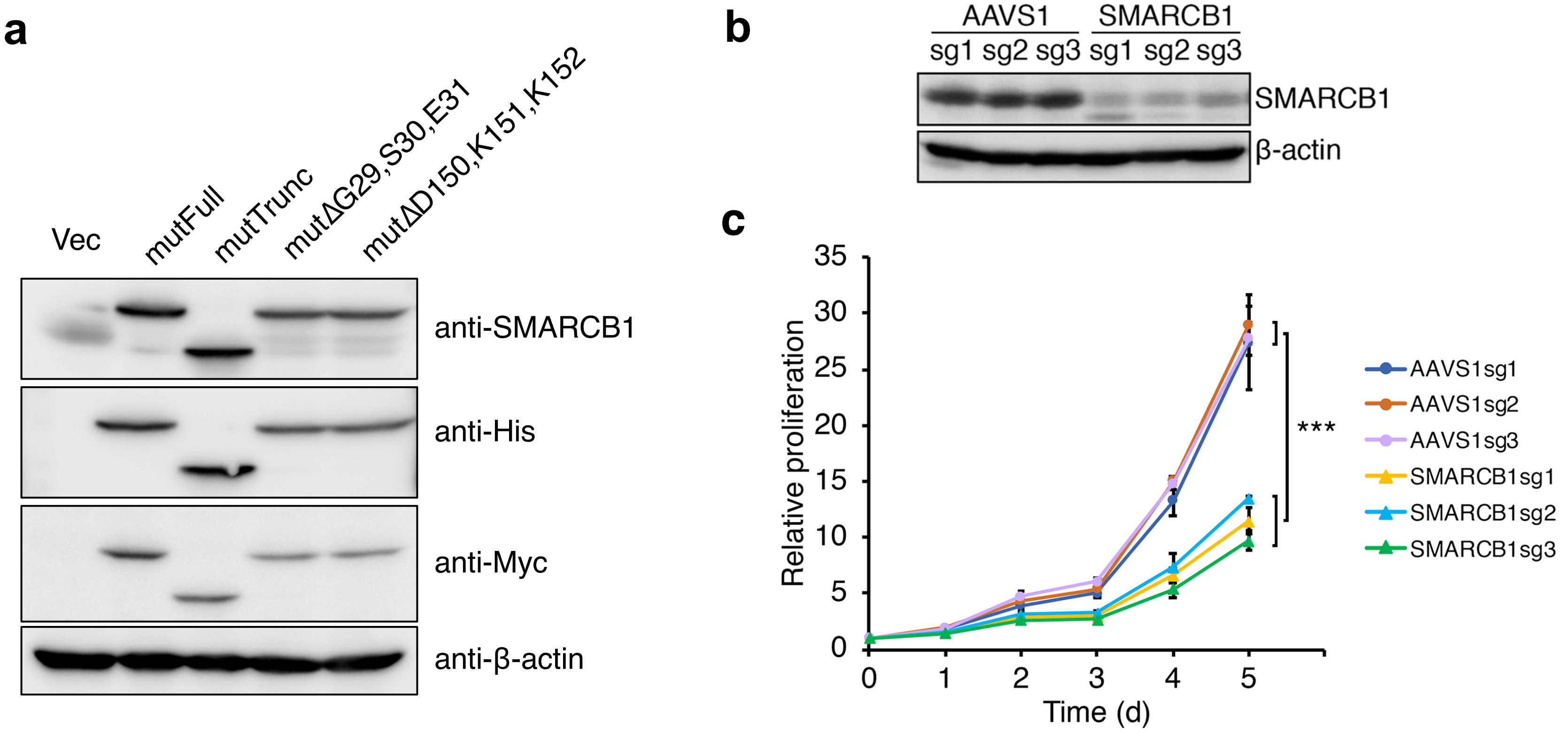

Supplementary Figure 6 a) Western blot showing the expression of exogenous mutant full-length, truncated, Δ29-31 and Δ150-152 forms of SMARCB1. **b)** Western blot showing knockout effect of three sgRNAs targeting *SMARCB1*. **c)** Relative proliferation of DLD-1 cells after SMARCB1 knockout by CRISPR/Cas9. The sgRNAs targeting the *AAVS1* locus were used as the controls. The error bars represent the standard divation of three biological replicates performed at each time point. The star symbols represent statistical significance: $p<0.001$ (***). The p-values were computed using t-test. Source data are provided as a Source Data file.

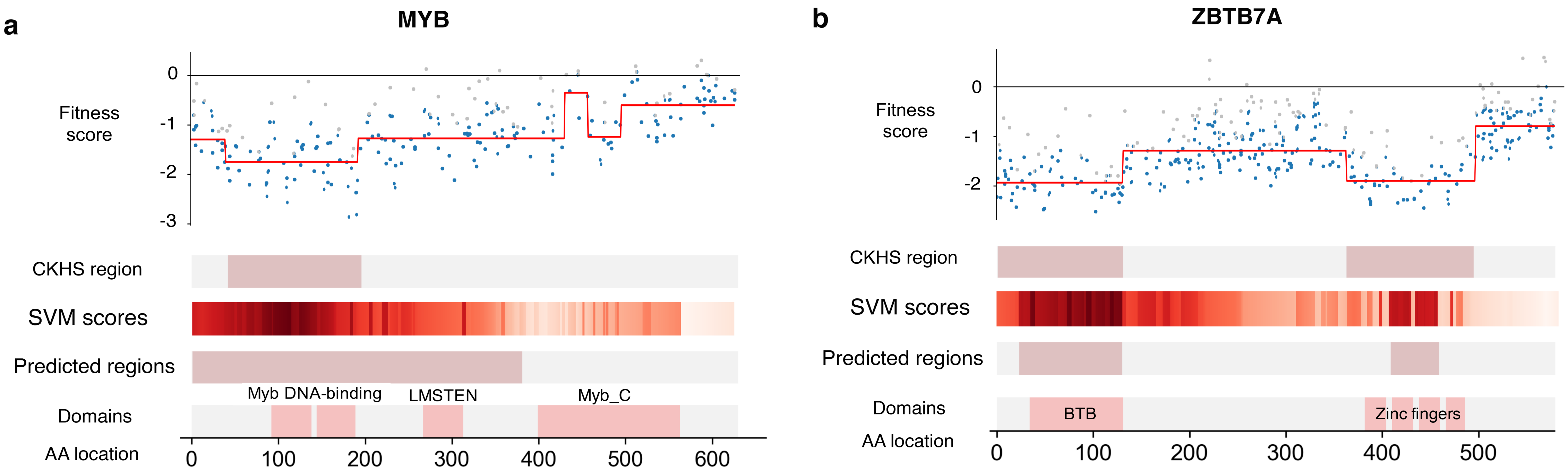

Supplementary Figure 7 The CKHS profile, SVM essential region prediction and domain annotation of **a)** MYB and **b)** ZBTB7A.

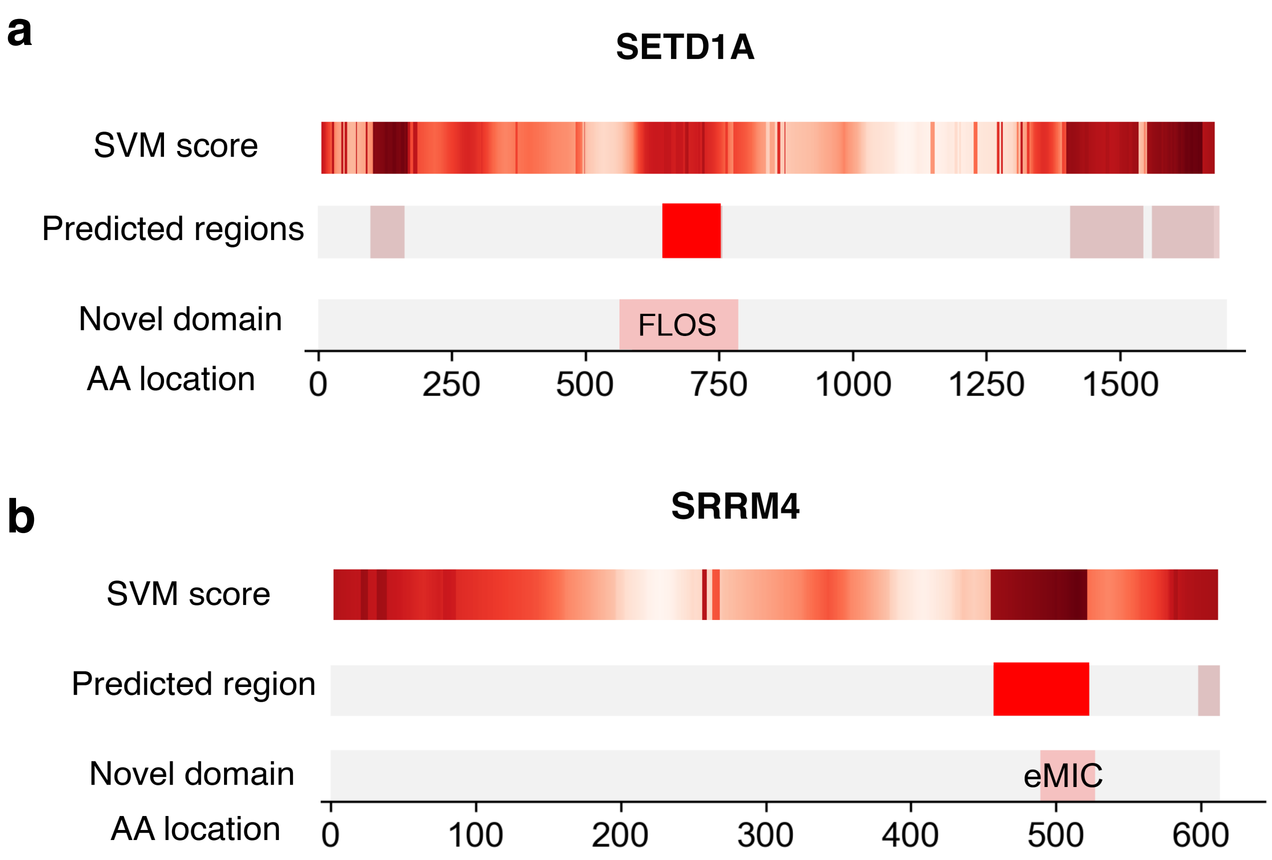

Supplementary Figure 8 The SVM predicted essential regions and newly identified functional domains [4,5] of **a)** SETD1A and **b)** SRRM4.

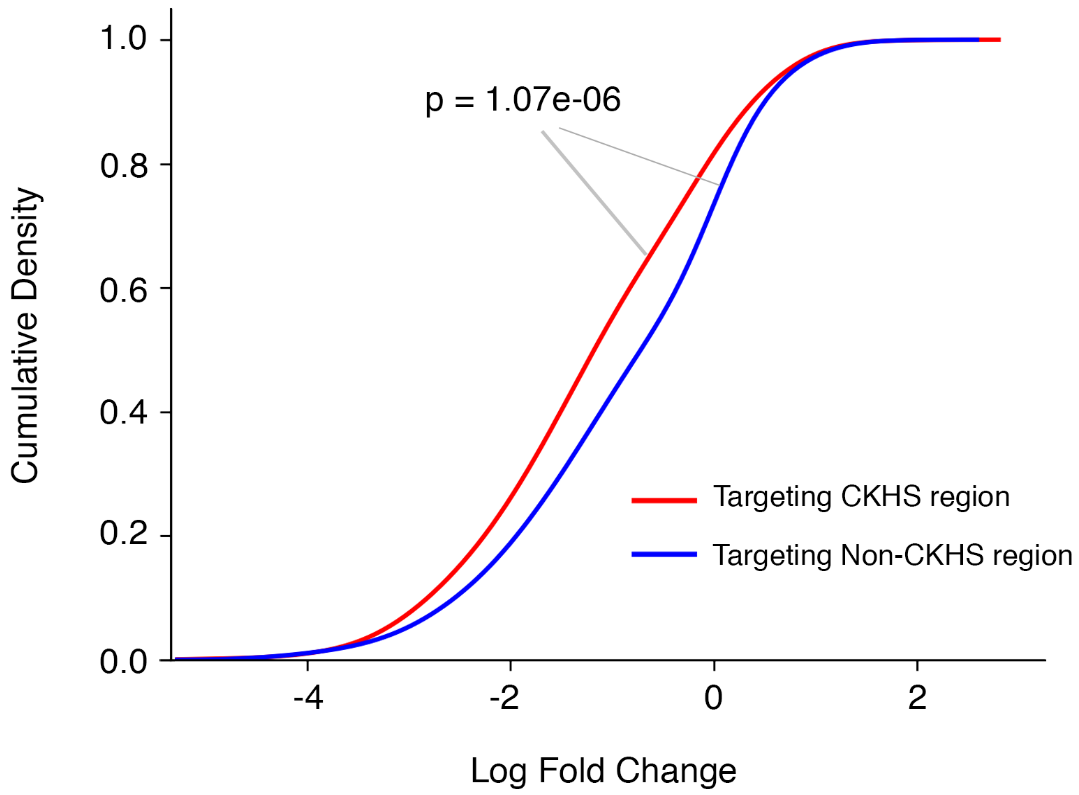

Supplementary Figure 9 Cumulative distribution of dropout effect (log fold change) for sgRNAs targeting CKHS region and Non-CKHS regions in GeCKO dataset [6]. The p-value was calculated using Kolmogrov-smirnov test.

a

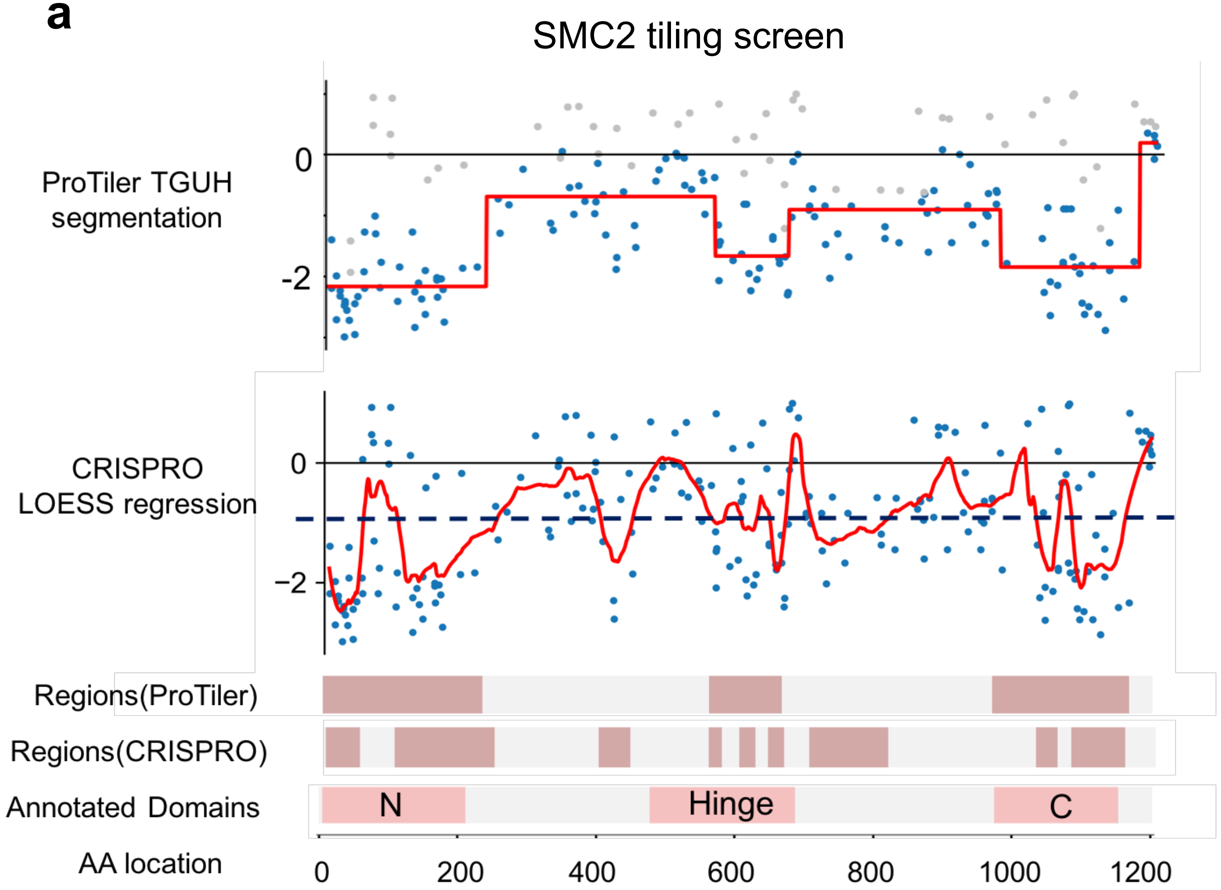

b

Metrics	ProTiler	CRISPRO
# Identified AAs	33827	33827
Precision	0.642	0.600
Recall	0.639	0.598
F1 score	0.640	0.599
# Identified regions	175	364
% Regions overlapped with Pfam domains	82.3%	62.3%
% Pfam domains identified	74.7%	78.3%
% Regions within 20 AAs from left borders of Pfam domains	46.4%	37.7%
% Regions within 20 AAs from right borders of Pfam domains	44.3%	39.0%

Supplementary Figure 10 a) The comparison between ProTiler and CRISPRO for detecting essential domains from tiling CRISPR screen data using SMC2 as an example. The threshold for CRISPRO was set allowing same number of amino acids within the CKHS regions called by CRISPRO and ProTiler. **b)** Quantitative comparison between ProTiler and CRISPRO at AA level (red) and region level (green).

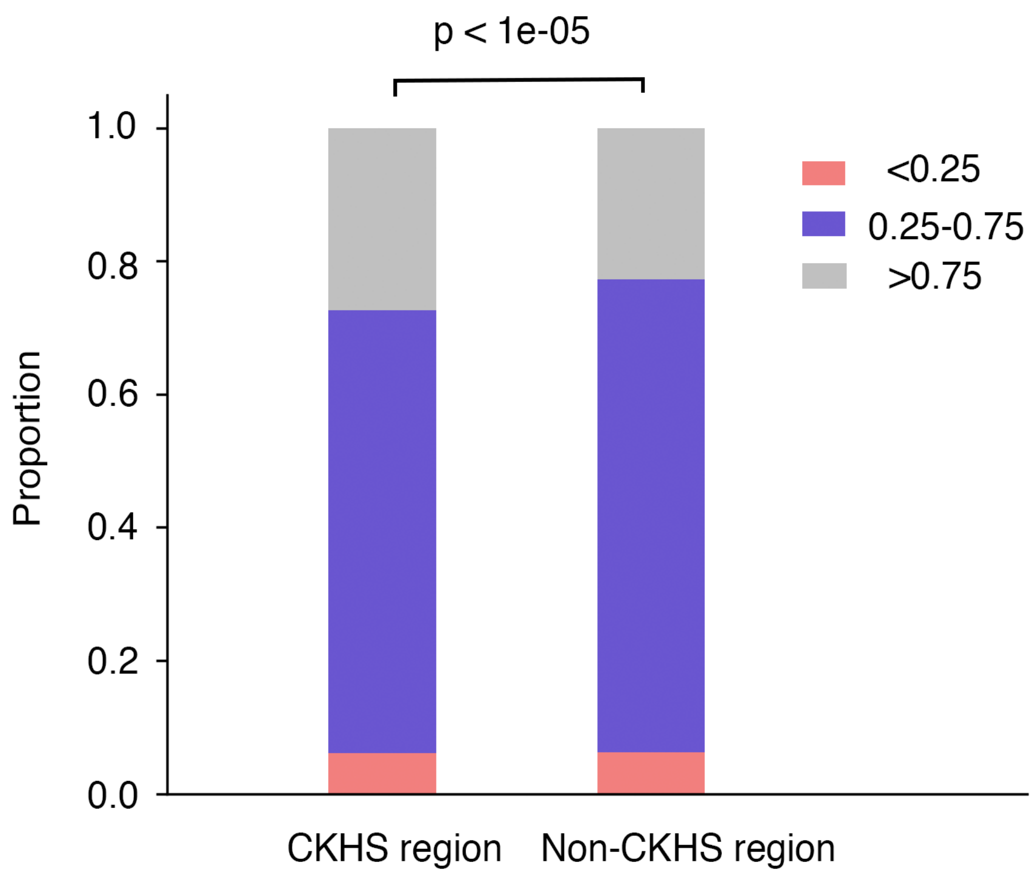

Supplementary Figure 11 Proportion of amino acids with different levels of transcripts coverage in CKHS region and Non-CKHS region. The p-value was calculated using Chi-square test.

References

1. Munoz, D.M. et al. CRISPR Screens Provide a Comprehensive Assessment of Cancer Vulnerabilities but Generate False-Positive Hits for Highly Amplified Genomic Regions. *Cancer Discov* **6**, 900-913 (2016).
2. Doench, J.G. et al. Optimized sgRNA design to maximize activity and minimize off-target effects of CRISPR-Cas9. *Nat Biotechnol* **34**, 184-191 (2016).
3. Xu, H. et al. Sequence determinants of improved CRISPR sgRNA design. *Genome Res* **25**, 1147-1157 (2015).
4. Torres-Mendez, A. et al. A novel protein domain in an ancestral splicing factor drove the evolution of neural microexons. *Nat Ecol Evol* **3**, 691-701 (2019).
5. Hoshii, T. et al. A Non-catalytic Function of SETD1A Regulates Cyclin K and the DNA Damage Response. *Cell* **172**, 1007-1021 e1017 (2018).
6. Aguirre, A.J. et al. Genomic Copy Number Dictates a Gene-Independent Cell Response to CRISPR/Cas9 Targeting. *Cancer Discov* **6**, 914-929 (2016)
