# Supplementary material for: De novo identification of essential protein domains from CRISPR-Cas9 tiling-sgRNA knockout screens
Source: Nat Commun. 2019 Oct 4;10:4541. doi: 10.1038/s41467-019-12489-8 (PMC6778102; doi:10.1038/s41467-019-12489-8)
Supplement: Supplementary file 7 — Description of Additional Supplementary Files [file 41467_2019_12489_MOESM7_ESM.pdf]

Title: Supplementary Data 1:
Description: Annotations of 15 essential protein domains targeted by small molecular drugs approved by FDA or in clinical trials.

Title: Supplementary Data 2:
Description: Averaged Z-scores of 159 genes in three different cell lines in the Munoz dataset, essentiality is assessed with threshold of -0.4.

Title: Supplementary Data 3:
Description: Annotation of 175 CKHS regions in 83 proteins called by ProTiler.

Title: Supplementary Data 4:
Description: Annotations of Pfam domains and their CKHS states for 51 multi-domain proteins in the Munoz dataset.

Title: Supplementary Data 5:
Description: Proteome-wide prediction of CKHS regions.
