# Supplementary material for: De novo identification of essential protein domains from CRISPR-Cas9 tiling-sgRNA knockout screens
Source: Nat Commun. 2019 Oct 4;10:4541. doi: 10.1038/s41467-019-12489-8 (PMC6778102; doi:10.1038/s41467-019-12489-8)
Supplement: Supplementary file 8 — Reporting Summary [file 41467_2019_12489_MOESM8_ESM.pdf]

nature research

Corresponding author(s): Han Xu

Last updated by author(s): Aug 21, 2019

Reporting Summary

Nature Research wishes to improve the reproducibility of the work that we publish. This form provides structure for consistency and transparency in reporting. For further information on Nature Research policies, see Authors & Referees and the Editorial Policy Checklist.

Statistics

For all statistical analyses, confirm that the following items are present in the figure legend, table legend, main text, or Methods section.

n/a	Confirmed	
[ ]	[x]	The exact sample size (*n*) for each experimental group/condition, given as a discrete number and unit of measurement
[ ]	[x]	A statement on whether measurements were taken from distinct samples or whether the same sample was measured repeatedly
[ ]	[x]	The statistical test(s) used AND whether they are one- or two-sided<br>*Only common tests should be described solely by name; describe more complex techniques in the Methods section.*
[ ]	[x]	A description of all covariates tested
[ ]	[x]	A description of any assumptions or corrections, such as tests of normality and adjustment for multiple comparisons
[ ]	[x]	A full description of the statistical parameters including central tendency (e.g. means) or other basic estimates (e.g. regression coefficient) AND variation (e.g. standard deviation) or associated estimates of uncertainty (e.g. confidence intervals)
[ ]	[x]	For null hypothesis testing, the test statistic (e.g. *F*, *t*, *r*) with confidence intervals, effect sizes, degrees of freedom and *P* value noted<br>*Give P values as exact values whenever suitable.*
[x]	[ ]	For Bayesian analysis, information on the choice of priors and Markov chain Monte Carlo settings
[x]	[ ]	For hierarchical and complex designs, identification of the appropriate level for tests and full reporting of outcomes
[ ]	[x]	Estimates of effect sizes (e.g. Cohen's *d*, Pearson's *r*), indicating how they were calculated

Our web collection on statistics for biologists contains articles on many of the points above.

Software and code

Policy information about availability of computer code

Data collection	NA
Data analysis	ProTiler was written in Python (version 2.7) and R package (version > 3.5.0), implemented as open source software downloadable from https://github.com/MDhewei/ProTiler-1.0.0 . It depends on Python packages including matplotlib version 2.2.3, pandas version 0.21.0, seaborn version0.8.0 and numpy version1.13.3 , and two R packages breakfast version 1.0.0 and stringr version1.4.0

For manuscripts utilizing custom algorithms or software that are central to the research but not yet described in published literature, software must be made available to editors/reviewers. We strongly encourage code deposition in a community repository (e.g. GitHub). See the Nature Research guidelines for submitting code & software for further information.

Data

Policy information about availability of data

All manuscripts must include a data availability statement. This statement should provide the following information, where applicable:

- Accession codes, unique identifiers, or web links for publicly available datasets
- A list of figures that have associated raw data
- A description of any restrictions on data availability

Accession numbers of all data analysed in this study are listed in this published article (and its supplementary information files.

Field-specific reporting

Please select the one below that is the best fit for your research. If you are not sure, read the appropriate sections before making your selection.

[x] Life sciences [ ] Behavioural & social sciences [ ] Ecological, evolutionary & environmental sciences

For a reference copy of the document with all sections, see nature.com/documents/nr-reporting-summary-flat.pdf

Life sciences study design

All studies must disclose on these points even when the disclosure is negative.

Sample size	Sample sizes are reported where statistic tests were performed
Data exclusions	All accession numbers of data included/excluded in this study are specified in the supplementary materials
Replication	Computational method was applied to publicly available data sets, so there are no replicates. All biological experiments were performed independently for three times.
Randomization	This is not relevant to our study in which we developed a computational method and applied it to publicly available dataset.
Blinding	This is not relevant to our study in which we developed a computational method and applied it to publicly available dataset.

Reporting for specific materials, systems and methods

We require information from authors about some types of materials, experimental systems and methods used in many studies. Here, indicate whether each material, system or method listed is relevant to your study. If you are not sure if a list item applies to your research, read the appropriate section before selecting a response.

Materials & experimental systems

n/a	Involved in the study	
[ ]	[x]	Antibodies
[ ]	[x]	Eukaryotic cell lines
[x]	[ ]	Palaeontology
[x]	[ ]	Animals and other organisms
[x]	[ ]	Human research participants
[x]	[ ]	Clinical data

Methods

n/a	Involved in the study	
[x]	[ ]	ChIP-seq
[x]	[ ]	Flow cytometry
[x]	[ ]	MRI-based neuroimaging

Antibodies

Antibodies used	anti-SMARCB1 (#A301-087A-M, Bethyl), anti-His (#12698T, CST), anti-Myc (#2278T, CST), anti-rabbit (#NA934, GE) and mouse (#NA931, GE)
Validation	All the antibodies in this study were used for Western blot to determine protein expression.

Eukaryotic cell lines

Policy information about cell lines

Cell line source(s)	Cell lines DLD-1 and HEK293T was obtained from ATCC.
Authentication	Authenticated as claimed by ATCC.
Mycoplasma contamination	Cell lines were tested to be mycoplasma-negative prior to performing the experiments.
Commonly misidentified lines<br>(See ICLAC register)	No commonly-misidentified lines were used in this study.
